# Extrasynaptic Localization Is Essential for α5GABA_A_ Receptor Modulation of Dopamine System Function

**DOI:** 10.1523/ENEURO.0344-23.2023

**Published:** 2024-03-12

**Authors:** Alexandra M. McCoy, Thomas D. Prevot, Md Yeunus Mian, Dishary Sharmin, Adeeba N. Ahmad, James M. Cook, Etienne L. Sibille, Daniel J. Lodge

**Affiliations:** ^1^Department of Pharmacology and Center for Biomedical Neuroscience, UT Health San Antonio, San Antonio, Texas 78229; ^2^South Texas Veterans Health Care System, Audie L. Murphy Division, San Antonio, Texas 78229; ^3^Campbell Family Mental Health Research Institute of CAMH, Toronto, Ontario M5G 2C1, Canada; ^4^Department of Psychiatry, University of Toronto, Toronto, Ontario M5S 1A1, Canada; ^5^Department of Chemistry and Biochemistry, University of Wisconsin-Milwaukee, Milwaukee, Wisconsin 53211; ^6^University of Texas, Rio Grande Valley, Edinburg, Texas 78539; ^7^Department of Pharmacology and Toxicology, University of Toronto, Toronto, Ontario M5S 1A1, Canada

**Keywords:** dopamine, electrophysiology, GABA, psychosis, radixin, rat

## Abstract

Dopamine system dysfunction, observed in animal models with psychosis-like symptomatology, can be restored by targeting gamma-aminobutyric acid type A receptors (GABA_A_Rs) containing the α5, but not α1, subunit in the ventral hippocampus (vHipp). The reason for this discrepancy in efficacy remains elusive; however, one key difference is that gamma-aminobutyric acid type A receptors containing the α1 subunit (α1GABA_A_Rs) are primarily located in the synapse, whereas gamma-aminobutyric acid type A receptors containing the α5 subunit (α5GABA_A_Rs) are mostly extrasynaptic. To test whether receptor location is responsible for this difference in efficacy, we injected an siRNA into the vHipp to knock down radixin, a scaffolding protein that holds α5GABA_A_Rs in the extrasynaptic space. We then administered GL-II-73, a positive allosteric modulator of α5GABA_A_Rs (α5-PAM) known to reverse shock-induced deficits in dopamine system function, to determine if shifting α5GABA_A_Rs from the extrasynaptic space to the synapse would prevent the effects of α5-PAM on dopamine system function. As expected, the knockdown of radixin significantly decreased radixin-associated α5GABA_A_Rs and increased the proportion of synaptic α5GABA_A_Rs, without changing the overall expression of α5GABA_A_Rs. Importantly, GL-II-73 was no longer able to modulate dopamine neuron activity in radixin-knockdown rats, indicating that the extrasynaptic localization of α5GABA_A_Rs is critical for hippocampal modulation of the dopamine system. These results may have important implications for clinical use of GL-II-73, as periods of high hippocampal activity appear to favor synaptic α5GABA_A_Rs; thus, efficacy may be diminished in conditions where aberrant hippocampal activity is present.

## Significance Statement

Currently available treatments for psychosis, a debilitating symptom linked with several brain disorders, are inadequate. While they can help manage symptoms in some patients, they do so imperfectly. They are also associated with severe side effects that can cause discontinuation of medication. This study provides preclinical evidence that the drug GL-II-73 possesses the ability to modulate dopamine activity, a key player in psychosis symptoms, and further provides some mechanistic details regarding these effects. Overall, this work contributes to the growing body of literature suggesting that GL-II-73 and similar compounds may possess antipsychotic efficacy.

## Introduction

Gamma-aminobutyric acid type A receptors containing the α5 subunit (α5GABA_A_Rs) have received considerable attention as a therapeutic target for multiple disorders involving hippocampal pathology, likely due to their enhanced expression within CA1 and CA3 regions of the hippocampus (Fritschy and Mohler, 1995; Sur et al., 1999; Olsen and Sieghart, 2009). Of particular interest to the pharmaceutical industry are positive allosteric modulators (PAMs) selective for α5GABA_A_Rs because of their low propensity for side effects compared with nonselective benzodiazepines, which are known to cause sedation through actions mediated by α1 subunits (Sieghart and Savić, 2018; Sigel and Ernst, 2018). Preclinical studies using positive allosteric modulators of α5GABA_A_Rs (α5-PAMs) have demonstrated a range of beneficial effects when given acutely including the following: anxiolytic, antidepressant, and procognitive effects (Prevot et al., 2019, 2020). When administered chronically, α5-PAMs can reverse stress- or age-related neuronal atrophy in the hippocampus and prefrontal cortex (Prevot et al., 2020; Bernardo et al., 2022). Additionally, α5-PAMs have shown promise in preclinical studies as antipsychotics (Gill et al., 2011; McCoy et al., 2022; Perez et al., 2022). These results suggest that α5-PAMs may have therapeutic utility for multiple disorders, especially those in which aberrant hippocampal activity is present.

Though the dopamine hypothesis asserts that psychosis is driven by excessive dopamine, convergent evidence suggests that dopamine dysregulation is secondary to aberrant hippocampal output, which drives dopamine system dysfunction (Lodge and Grace, 2007, 2008b, 2011). Indeed, increased hippocampal activity has been observed in humans with psychosis (Schobel et al., 2009) and in rodent models (Lodge and Grace, 2007, 2008b, 2011). Further, we have previously demonstrated that decreasing hippocampal activity using pharmacological (Perez and Lodge, 2018), cell-based (Perez and Lodge, 2013; Donegan et al., 2017), or surgical (Perez et al., 2013) approaches effectively normalizes dopamine system function and related behaviors in rodent models with schizophrenia-like symptomatology. Thus, we posit that augmenting the function of α5GABA_A_Rs in the ventral hippocampus (vHipp) will likely have the same effect and normalize dopamine system function and behavior in a stress-based model displaying psychosis-like pathology. Indeed, we have previously demonstrated that this is the case in animal models used to study both schizophrenia and posttraumatic stress disorder (PTSD) where aberrant dopamine system function is present (Donegan et al., 2019; McCoy et al., 2022; Perez et al., 2022). This evidence suggests that α5GABA_A_Rs may represent a viable therapeutic target for the treatment of psychosis across multiple disorders.

**Figure 1.**
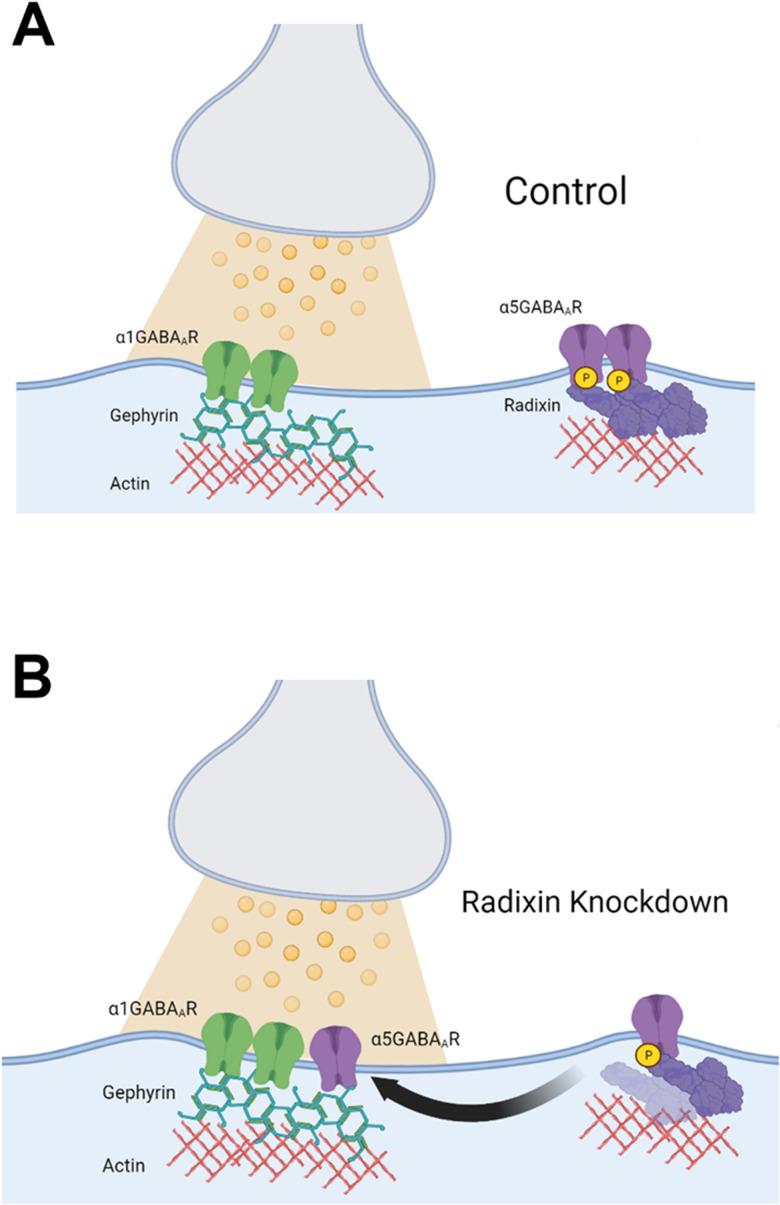
Schematic of radixin knockdown. Diagram of an inhibitory synapse and surrounding extrasynaptic area under (***A***) baseline conditions and (***B***) when radixin is knocked down. Figure made using BioRender.

Interestingly, nonspecific positive allosteric modulation of gamma-aminobutyric acid type A receptors (GABA_A_Rs) or selectively targeting hippocampal gamma-aminobutyric acid type A receptors containing the α1 subunit (α1GABA_A_Rs) are largely ineffective at reversing aberrant dopamine system function (Donegan et al., 2019; Perez et al., 2022). One crucial difference between α5- and α1GABA_A_Rs is that α5GABA_A_Rs can dynamically travel between the extrasynaptic spaces, where they regulate tonic inhibition (Caraiscos et al., 2004; Bonin et al., 2007; Glykys et al., 2008) and the synapse, where they mediate phasic inhibition (Davenport et al., 2021), whereas α1GABA_A_Rs are almost exclusively synaptic (Brünig et al., 2002; Crestani et al., 2002). Unlike typical extrasynaptic receptors that are diffused in the membrane, α5GABA_A_Rs form clusters (Brünig et al., 2002; Loebrich et al., 2006; Hausrat et al., 2015). This clustering is mediated through an interaction with radixin, a scaffolding protein that anchors the receptor to actin, concentrating receptors in the extrasynaptic space (Fig. 1; Loebrich et al., 2006). The radixin–α5 interaction is phosphorylation-dependent, such that dephosphorylation of radixin decouples the two proteins, allowing the receptor to diffuse freely through the membrane (Hausrat et al., 2015). In mutant mice that express a phosphorylation-incompetent radixin, α5GABA_A_Rs colocalize with gephyrin, the inhibitory synaptic scaffolding protein, suggesting that, in the absence of a radixin interaction, α5GABA_A_Rs will move into the synapse and interact with gephyrin (Loebrich et al., 2006; Hausrat et al., 2015). Furthermore, the shifting of α5GABA_A_Rs into the synapse appears to be physiologically relevant, as induction of long-term potentiation in the hippocampus can also increase synaptic relocalization of α5GABA_A_Rs (Davenport et al., 2021). Indeed, it has been hypothesized that the purpose of α5GABA_A_Rs clustering is to serve as a readily releasable pool of GABA_A_Rs to rapidly adjust to perturbations of excitatory/inhibitory balance, with periods of high activity increasing the contribution of α5GABA_A_Rs to inhibitory postsynaptic potentials (Davenport et al., 2021).

Given the remarkable difference in antipsychotic-like efficacy between targeting α5- and α1GABA_A_Rs (Donegan et al., 2019; Perez et al., 2022), we sought to examine if the receptor location (extrasynaptic vs synaptic) of α5GABA_A_Rs could influence the effects of a selective α5-PAM, GL-II-73, on dopamine system function and sensorimotor gating [prepulse inhibition (PPI) of startle], a dopamine-dependent behavior often affected in psychosis (Swerdlow et al., 2001). We injected siRNA targeting radixin or a scrambled siRNA, as a control, directly into the vHipp of adult rats. Under these conditions, we examined the effects of GL-II-73 on dopamine neuron population activity in the ventral tegmental area (VTA) and on PPI. The exposure to an inescapable shock (IS) for 2 d is a validated model used to study PTSD-like pathology in rodents (Van Dijken et al., 1992), a condition often comorbid with psychosis (Compean and Hamner, 2019). In this model, we have demonstrated that the rats exhibit psychosis-like symptomatology, such as robust alterations in dopamine neuron activity and deficits in PPI (Elam et al., 2021) that can be reversed by GL-II-73 (McCoy et al., 2022). Here, we now report that this reversal was blunted following radixin knockdown, causing α5GABA_A_Rs to shift into the synapse. These findings establish a clear relationship between α5GABA_A_R localization and the antipsychotic-like efficacy of GL-II-73. Such information is critical for the clinical use of α5-PAMs, as the α5GABA_A_R location appears to be activity dependent (Davenport et al., 2021), and conditions in which hippocampal hyperactivity is present (e.g., epilepsy-induced psychosis) may promote a synaptic shift of α5GABA_A_Rs and would decrease antipsychotic efficacy in these individuals.

## Materials and Methods

All experiments were performed in accordance with the guidelines outlined in the United States Public Health Service Guide for the Care and Use of Laboratory Animals and were approved by the Institutional Animal Care and Use Committees of UT Health San Antonio and United States Department of Veterans Affairs.

### Animals

Adult male (350–400 g) and female (250–300 g) Sprague Dawley rats purchased from Envigo were used for all experiments. Rats were kept on a 12 h light/dark cycle. Food and water were provided *ad libitum*. GL-II-73 or vehicle (85% H_2_0, 14% propylene glycol, 1% Tween 80) were administered directly into the vHipp (100 ng/µl; 0.75 µl; *A*/*P* −5.3 mm, ML ± 5.0 mm, from the bregma; *D*/*V* −6.0 mm from the brain surface) at a rate of approximately 0.5 μl/min 20 min prior to electrophysiology or behavior. This dose and timing were selected based on previous characterization (Prevot et al., 2019) as well as our own data (McCoy et al., 2022; Perez et al., 2022).

### siRNA-mediated knockdown of radixin

Rats were anesthetized with 2–4% isoflurane prior to placement in a stereotaxic apparatus using blunt atraumatic ear bars. Bilateral indwelling cannulas (Protech International, C317G, *D*/*V* −6 mm below plate) were implanted in the vHipp (*A*/*P* −5.3 mm *M*/*L* ± 5.0 mm from the bregma; *D*/*V* −6.0 mm from the brain surface) and fixed in place with dental cement and four anchor screws. Rats received the analgesic ketoprofen (5 mg/kg, s.c.) and were allowed to recover, individually housed, for a minimum of 1 week before experimentation. Injectors extending 1 mm past the end of the guide cannula were utilized for microinjections. Guide cannulas were kept patent with dummy cannulas. Rats were injected with either siRNA targeting radixin (0.2 µg/µl; 0.75 µl) or a nontargeting, scrambled siRNA as a control at a rate of approximately 0.5 µl/min. This concentration was selected based on published data (Mitchnick et al., 2015). The four radixin targeting sequences in the siRNA SMARTpool are as follows: GAAUCAGUUAUAACGUUUA; CCAAUAAAUGUAAGAGUAA; CCUUAUUGCUAAAAGAAUC; and CUCUAAUUUUGGAUAAUAU. Accell siRNA (Dharmacon) was chosen specifically as it was designed to incorporate into cells that are difficult to transfect, such as neurons, without the use of a transfection agent and results in the peak knockdown within 3–4 d (Webb et al., 2017; Jarome et al., 2018).

### Inescapable footshock stress

Rats were randomly assigned to the control (no shock) or to the shock groups that received 2 consecutive days of inescapable footshock stress as previously described (Elam et al., 2021; McCoy et al., 2022). The 2 d IS paradigm consisted of placing the rats in a 30.5 × 25.4 × 30.5 cm conditioning chamber with a stainless steel grid shock floor (Coulbourn Instruments). One session of IS consists of 60 × 15 s, 0.8 mA footshocks with an average intertrial interval (ITI) of 30 s with a 25% deviation (±7.5 s) and lasted approximately 40 min. Control rats were handled daily but not exposed to conditioning chambers. Electrophysiology and behavioral experiments were conducted 24 h after the last day of IS as previously described (Elam et al., 2021; McCoy et al., 2022).

### In vivo extracellular dopamine recordings

Rats were anesthetized with 8% chloral hydrate (400 mg/kg, i.p.) and placed in a stereotaxic apparatus (Kopf Instruments). This anesthetic was specifically chosen as it does not significantly alter dopamine neuron activity compared with freely moving animals(Hyland et al., 2002) and also produces analgesia (Ward-Flanagan and Dickson, 2023). Extracellular glass microelectrodes (impedance, ∼6–10 MΩ) were lowered into the VTA (from the bregma: *A*/*P* −5.3 mm, *M*/*L* ±0.6 mm, and *D*/*V* −6.5 to −9.0 mm) using a hydraulic micropositioner (model 640, Kopf Instruments). Multiple areas within the VTA were sampled by making multiple vertical passes (“tracks”), separated by 200 µm, in a predetermined pattern. Spontaneously active dopamine neurons within a track were identified using open filter settings (low-frequency cutoff, 30 Hz; high-frequency cutoff, 30 kHz) according to previously established electrophysiological criteria (Grace and Bunney, 1983; Ungless and Grace, 2012). Three parameters of dopamine activity were measured and analyzed: (1) the number of dopamine neurons firing spontaneously per track (population activity; Lodge and Grace, 2011), (2) firing rate, and (3) proportion of action potentials occurring in bursts (defined as the incidence of spikes with <80 ms between them; termination of the burst is defined by >180 ms between spikes). The analysis of dopamine neuron activity was performed using the LabChart software (ADInstruments). Shortly after, rats were rapidly decapitated, and brains were extracted. A subset of brains was used to verify electrode and cannula placement, while the remaining brains were used for molecular analysis.

### Prepulse inhibition of the startle response

Rats were placed into a sound-attenuated chamber (San Diego Instruments) and allowed to acclimate to 65 dB background noise for 5 min. Rats were then exposed to 10 startle-only trials (40 ms, 120 dB, 15 s average ITI). Next, rats were exposed to 24 trials where a prepulse (20 ms at 69 dB, 73 dB, and 81 dB) was presented 100 ms before the startle pulse. Each prepulse and startle pulse trial was presented six times in a pseudorandom order (15 s average ITI). The startle response was measured from 10 to 80 ms after the onset of the startle pulse and recorded and analyzed using the SR-LAB Analysis Software (San Diego Instruments). PPI was calculated for each prepulse intensity and averaged across the three intensities.

### Immunoprecipitation

Immediately following completion of electrophysiology or behavior, rats were killed by rapid decapitation, and the hippocampus was dissected out on ice and separated into dorsal and ventral portions. Samples were homogenized with lysis buffer and centrifuged at 14,000 × *g* for 2 min. Supernatants were collected and stored at −80 until α5 subunit, and its binding partners were immunoprecipitated using SureBeads Protein G Magnetic Beads according to the manufacturer's protocol (Bio-Rad). Western blots (detailed in Western blot) were used to quantify α5, radixin, and gephyrin levels in both the immunoprecipitated samples and hippocampal homogenates.

### Chemical cross-linking assay

A chemical cross-linking assay was performed in a subset of rats as previously described (Boudreau et al., 2012; Tomoda et al., 2022). Briefly, rats were rapidly decapitated, and brains were extracted. The whole hippocampus was dissected out on ice, separated into dorsal and ventral portions, and minced into small pieces using a razor blade. The vHipp from one hemisphere was incubated in Dulbecco's phosphate-buffered saline (PBS) with calcium chloride and magnesium chloride (Sigma-Aldrich) containing bis(sulfosuccinimidyl)suberate (BS3) cross-linker (2 mM, Thermo Fisher Scientific) for 2 h at 4C on a shaker. The other hemisphere was incubated in Dulbecco's PBS as a control. All samples were quenched by adding 100 mM glycine and rotating another 10 min at 4C. Samples were then centrifuged (20,000 × *g* for 2 min at 4C), and supernatants were discarded. A lysis buffer containing 0.1% Triton X-100 and peptidase inhibitors was added, and tissues were homogenized (PowerGen 125, Thermo Fisher Scientific) and centrifuged for 2 min (20,000 × *g* at 4C). The supernatants were collected and stored at −80 until analyses by Western blot.

### Western blot

Proteins in the lysates were separated in a sodium dodecyl–sulfate polyacrylamide gel electrophoresis followed by blotting onto a 0.2 µm nitrocellulose membrane. Membranes were incubated with an antibody against α5GABA_A_Rs (1:1,000), radixin (1:1,000), gephyrin (1:3,000), or GAPDH (1:1,000) in 2.5% BSA in TBST, overnight at 4C. They were then washed with Tris-buffered saline with 0.1% Tween 20 (TBST) prior to incubation with a horseradish peroxidase-conjugated secondary antibody (goat anti-rabbit, 1:10,000, horse anti-mouse, 1:5,000) for 1 h at room temperature. Membranes were washed with TBST (three times for 10 min each) and incubated with a Pierce enhanced chemiluminescence kit (Thermo Fisher Scientific) followed by exposure to x-ray film for detection. Blots were stripped using a commercially available stripping buffer, washed, blocked, and reprobed no more than once. Densitometry analyses of immunoreactive bands were performed using the NIH ImageJ software from the scanned films. Densitometric arbitrary units were normalized to GAPDH, except in immunoprecipitation studies in which radixin and gephyrin measures were normalized to α5GABA_A_R levels.

### Histology

**Figure 2.**
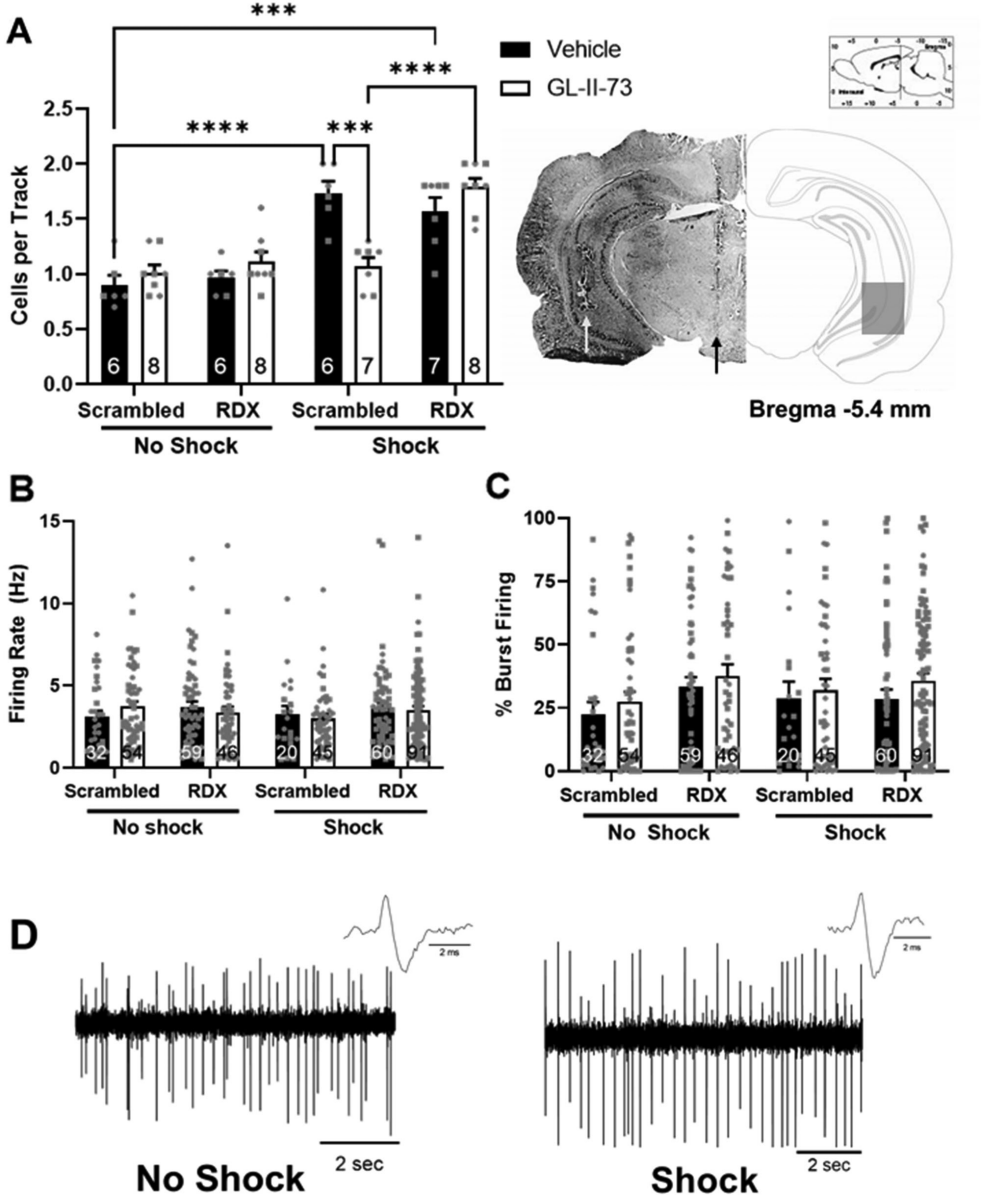
GL-II-73 was unable to restore dopamine system function when radixin was knocked down. In vivo extracellular electrophysiology was used to measure dopamine cell activity in the VTA. ***A***, left, IS exposure significantly increased the number of spontaneously active cells/track (population activity), which was reversed by intra-ventral hippocampus injection of GL-II-73 (100 ng/μl; 0.75 μl), but not when radixin was knocked down. Right, Representative brain slice with electrode placement in the VTA (black arrow) and cannula placement for drug administration in the vHipp (white arrow), with the corresponding schematics of the brain section (−5.40 mm posterior to bregma) with the box indicating the area in which tracks were found. Neither (***B***) firing rate nor (***C***) burst firing was affected by siRNA, shock, or drug treatment. ***D***, Representative traces from control (left) and shocked (right) rats. *n* = 6–8/group, males and females represented as circles and squares, respectively, ***p = 0.0001, ****p < 0.0001; RDX, radixin.

To verify electrode and cannula placement, we fixed brains for at least 24 h (4% phosphate-buffered formaldehyde) and cryoprotected (10% w/v sucrose in PBS) until saturated. Brains were coronally sectioned (25 µm) using a cryostat (Leica). Sections containing electrode or cannula tracks were mounted onto gelatin-coated slides, stained with neutral red (0.1%) and thionin acetate (0.01%), and coverslipped with DPX Mountant for histochemical confirmation within the VTA (electrode) or vHipp (cannula; Fig. 2*A*).

### Materials

The proprietary compound, GL-II-73, was synthesized by the University of Wisconsin–Milwaukee and supplied by the Centre for Addiction and Mental Health, Campbell Family Mental Health Research Institute. Chloral hydrate (C8383), propylene glycol (P4347), and Tween 80 (P1754) were obtained from Sigma-Aldrich. Antibodies were from R&D Systems, #PPS027 (α5); Abcam, ab5249 (radixin), ab181382 (gephryin), #9484 (GAPDH); or Cell Signaling Technology, #7074 (anti-rabbit HRP) and #7076 (anti-mouse HRP). Accell siRNA was purchased from Dharmacon.

### Statistical analysis

The data are represented as mean ± SEM and *n* values representing either the number of rats or neurons as indicated. In all experiments, the data were analyzed by three-way ANOVA (electrophysiology and PPI; factors, stress, drug, and siRNA), two-way ANOVA (α5 surface expression; factors, siRNA and cross-linker), or *t* test (Western blot) and plotted using Prism Software (GraphPad Software). When significant main effects or interactions were detected, the Holm–Sidak post hoc test was used. All tests were two-tailed, and significance was determined at *p* < 0.05. While both sexes were represented, we were not powered to detect sex differences and therefore did not explicitly test for this. Raw electrophysiology data were analyzed using LabChart version 8 (ADInstruments), and PPI data were analyzed using the SR-LAB Analysis Software (San Diego Instruments).

## Results

### The therapeutic effects of intra-vHipp administration of GL-II-73 on dopamine system function are blocked by radixin knockdown

To evaluate dopamine system function, we measured dopamine neuron activity in the VTA using in vivo extracellular electrophysiology. Consistent with previous findings (Elam et al., 2021; McCoy et al., 2022), inescapable footshock stress elicited a significant increase in the population activity (*n* = 6 rats; 1.733 ± 0.109 cells per track; three-way ANOVA; *F*_Shock (1,48) _= 73.860; *p* < 0.0001; *F*_siRNA (1,48)_ = 8.135; *p* = 0.006; Holm–Sidak; *t* =  6.153, *p* < 0.0001; Fig. 2*A*) when compared with nonshocked vehicle rats (*n* = 6 rats; 0.900 ± 0.089 cells per track). This shock-induced increase in dopamine neuron activity was completely reversed by the intra-hippocampal administration of GL-II-73 (*n* = 7 rats; 1.071 ± 0.078 cells per track; Holm–Sidak; *t *= 5.072, *p* = 0.0001) and had no effect in nonshocked rats who received intra-hippocampal GL-II-73 (*n* = 8 rats; 1.013 ± 0.069 cells per track). Further, knocking down radixin in nonshocked rats had no effect on population activity in the vehicle (*n* = 6 rats; 0.967 ± 0.061 cells per track) and GL-II-73-treated rats (*n* = 8 rats; 1.113 ± 0.088 cells per track). Again, consistent with observations in rats who received the scrambled siRNA, shock produced a significant increase in dopamine neuron activity (*n* = 7 rats; 1.571 ± 0.121 cells per track; Holm–Sidak; *t *= 1.780, *p* = 0.669) in radixin knockdown rats. Interestingly, increasing synaptic α5GABA_A_R localization by knocking down radixin blocked the ability of intra-hippocampal GL-II-73 to restore dopamine system function in shocked rats (*n* = 8 rats; 1.788 ± 0.081 cells per track). As expected, no significant differences were observed in the average firing rate (Fig. 2*B*; *F*_Shock (1,404) _= 0.953; *p *= 0.330; *F*_siRNA (1,404) _= 0.304; *p *=  0.582 *F*_drug (1,404)_ = 0.033; *p *=  0.856) or burst firing (Fig. 2C; *F*_Shock (1,404) _= 3.427; *p *= 0.065; *F*_siRNA (1,404)_ = 0.044; *p* = 0.833; *F*_drug (1,404)_ = 2.200; *p *=  0.139). Representative traces from control (left) and shock (right) animals are shown in Figure 2*D*.

### Increased synaptic α5GABA_A_R expression does not prevent the effects of GL-II-73 in the PPI of startle

**Figure 3.**
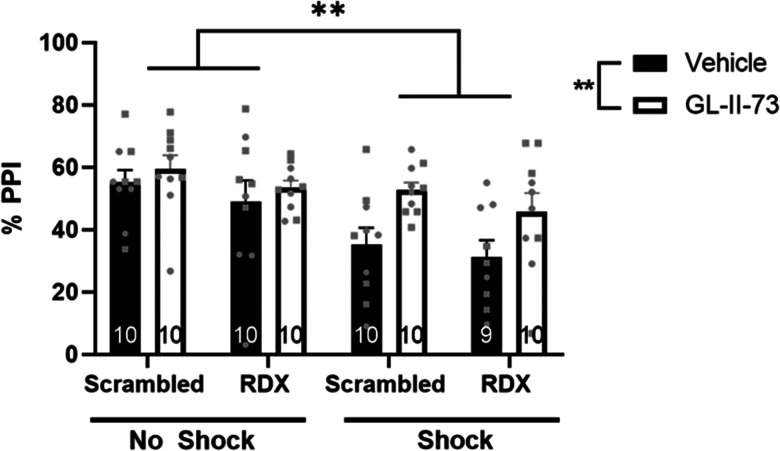
Radixin knockdown does not alter PPI. Two days of IS had a significant main effect on PPI as did treatment with GL-II-73 (*p* = 0.0042); however, post hoc analysis revealed no relevant group differences. *n* = 9–11/group, males and females represented as circles and squares, respectively. **P < 0.005; RDX, radixin.

To evaluate sensorimotor gating, we measured the PPI of the acoustic startle response (Fig. 3). Previous studies measuring PPI in rats exposed to IS reported a significant decrease in the %PPI following the inescapable footshock stress (Elam et al., 2021; McCoy et al., 2022). Here, we observed a significant main effect of shock (*n* = 9–11 rats per group; three-way ANOVA; *F*_Shock__(1,71) _= 14.310; *p* = 0.0003) and of intra-vHipp administration of GL-II-73 (*F*_drug__(1,71) _= 8.765; *p *= 0.004); however, post hoc tests revealed no significant differences between groups.

### Radixin knockdown increased markers of synaptic α5GABA_A_R but did not alter α5GABA_A_R expression within the vHipp

**Figure 4.**
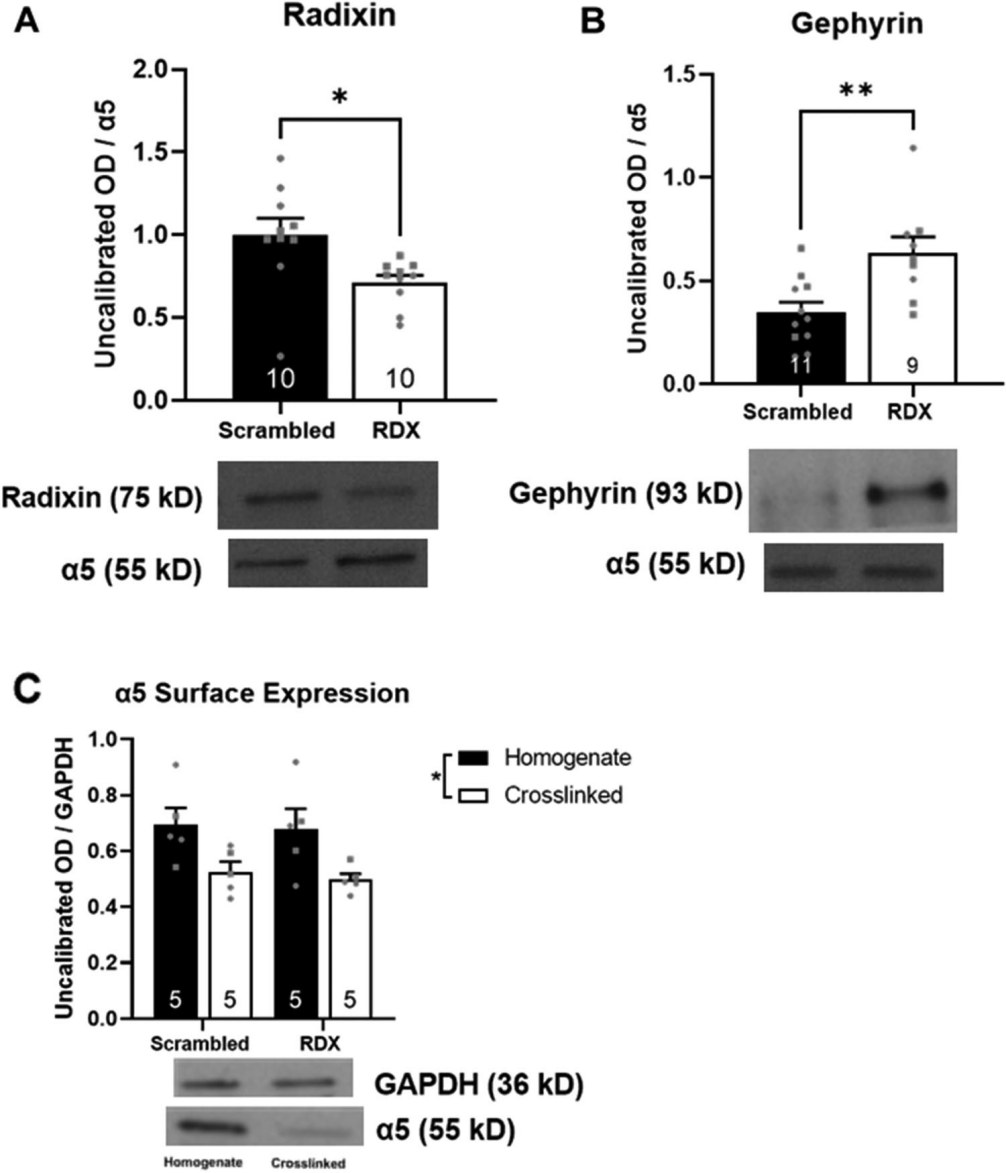
Radixin knockdown increases synaptic α5, without changing surface or total α5 expression. ***A***, Coimmunoprecipitation of α5 and radixin revealed a significant decrease in α5-associated radixin in rats that received radixin-targeted siRNA compared with those that received scrambled siRNA. Representative image of bands below. *n* = 10/group. ***B***, Conversely, coimmunoprecipitation of α5 and gephyrin revealed a significant increase in gephyrin levels in the radixin knockdown group. Representative image of bands below. *n* = 9–11/group. ***C***, Treatment with the cross-linking agent caused a significant decrease in the optical density of α5 immunoreactive bands, but no differences in total α5 (homogenate) or surface (cross-linked) were observed between rats that received scrambled siRNA or radixin-targeted siRNA. Representative image of bands below graphs. *n* = 5/group, males and females represented as circles and squares, respectively. *p < 0.05, **p < 0.01 *n* = 10; RDX, radixin; OD, optical density.

To validate the successful knockdown of radixin, we measured radixin associated with α5GABA_A_R using coimmunoprecipitation in control rats. We observed a significant difference between radixin knockdown and control groups (Fig. 4*A*, *t* test; *t *= 2.629, *p *= 0.017). Additionally, coimmunoprecipitation of α5GABA_A_R and gephyrin revealed a significant increase in gephyrin levels in rats that had radixin knocked down compared with controls (Fig. 4*B*, *t* test; *t *= 3.069, *p *= 0.008), suggesting an increase in synaptic α5GABA_A_Rs. Finally, to ensure any electrophysiology and behavioral results were not due to degradation or internalization of α5GABA_A_Rs when radixin is knocked down, we also measured surface and total α5GABA_A_R using a chemical cross-linking assay. While there was an expected significant difference between cross-linked samples and homogenate (Fig. 3*C*, two-way ANOVA, *F*_cross-linker (1,16) _= 11.300; *p *= 0.004), there were no significant differences due to radixin knockdown (*F*_siRNA_ _(1,16) _= 0.173; *p *= 0.683).

## Discussion

Psychosis is a debilitating symptom that accompanies many neurological disorders, including PTSD (van den Berg et al., 2016; Compean and Hamner, 2019). The dopamine hypothesis states that aberrant dopamine neuron activity underlies psychosis symptoms, yet currently available antipsychotics that target dopamine D2 receptors are not always effective and often result in intolerable side effects (i.e., dyskinesias and metabolic disorders; Lieberman et al., 2005). This has led some to suggest that indirectly modulating dopamine neuron activity through manipulating activity in upstream brain regions may be an effective treatment strategy that produces fewer adverse effects. The hippocampus is a brain region that can modulate dopamine neuron activity through a multisynaptic pathway starting in the nucleus accumbens (Lodge and Grace, 2007, 2008b). Using a multitude of techniques, we and others have demonstrated that attenuating vHipp activity can restore dopamine neuron population activity and related behaviors in animal models used to study psychosis (Lodge and Grace, 2007; Valenti et al., 2011; Perez et al., 2013; Perez and Lodge, 2013, 2018). An effective and translational approach to inhibiting vHipp activity is by using α5-PAMs. Indeed, we and others have previously shown that targeting α5GABA_A_Rs can improve physiological and behavioral alterations associated with psychosis (Gill et al., 2011; Donegan et al., 2019; McCoy et al., 2022; Perez et al., 2022), suggesting that α5-PAMs may possess antipsychotic efficacy. Interestingly, the efficacy of PAMs appears to be specific to those selective for the α5-subunit, as targeting vHipp α1GABA_A_Rs does not appear to modulate VTA dopamine neuron activity (Donegan et al., 2019; Perez et al., 2022). A major delineation between these receptor types is their cellular location, with α5GABA_A_Rs existing both in the synapse and extrasynaptic space, whereas α1GABA_A_Rs are limited to the synapse. Here, we examined if the observed differences in antipsychotic-like efficacy were due to receptor location (extrasynaptic vs synaptic). Based on our previous findings on targeting synaptic α1GABA_A_Rs, we posited that GL-II-73 would no longer be able to modulate dopamine neuron activity when α5GABA_A_Rs are shifted into the synapse. These studies have important implications, as previous studies have determined that high periods of hippocampal activity, often observed in psychosis (Lodge and Grace, 2007, 2008b; Schobel et al., 2009), can promote movement of α5GABA_A_Rs into the synapse.

Aberrant dopamine neuron activity is central to the pathology of psychosis and is observed in both patients (Laruelle and Abi-Dargham, 1999; Abi-Dargham, 2004; Howes et al., 2009) and rodent models (Lodge, 2013). To examine dopamine system function, we used in vivo electrophysiology to measure the number of spontaneously active dopamine neurons in the VTA, referred to as population activity (Lodge and Grace, 2011). We consistently find that animal models used to study psychosis have elevated dopamine neuron activity (Perez et al., 2013; Donegan et al., 2017; Perez and Lodge, 2019). Here, we report that IS exposure, a common rodent model to study PTSD, induced aberrant dopamine neuron population activity, a finding consistent with previous literature (Elam et al., 2021; McCoy et al., 2022). This was reversed by GL-II-73. However, in conditions of knocking down radixin, which caused a shift of α5GABA_A_Rs to the synapse, this effect of GL-II-73 was lost. These results suggest that the ability of GL-II-73 to modulate VTA dopamine neuron activity is dependent on the extrasynaptic localization of α5GABA_A_Rs.

Patients with PTSD and patients with psychosis both display deficits in sensorimotor gating (Bakshi et al., 2012; Kohl et al., 2013; Meteran et al., 2019), a behavioral dimension that is readily assessed in rodents using PPI (Braff and Geyer, 1990). Indeed, rodent models used to study both PTSD and psychosis display deficits in PPI (Perez et al., 2019; Elam et al., 2021), which can be reversed by GL-II-73 (McCoy et al., 2022). In the current study, we demonstrated that IS decreases PPI and that intervention with GL-II-73 attenuates this, regardless of the radixin knockdown (i.e., regardless of the localization of α5GABA_A_Rs). While PPI is a dopamine-dependent behavior, it is mediated by other circuits and not exclusively controlled by dopamine (Swerdlow et al., 2001). This may suggest that the reliance of GL-II-73 on extrasynaptic receptors is limited to modulation of dopamine neuron activity, and may not apply to the antidepressant-like effects (Prevot et al., 2019) or the procognitive effects (Prevot et al., 2020).

Nonselective benzodiazepines, or derivatives that primarily act on α1GABA_A_Rs, are ineffective as antipsychotics (Easton and Janicak, 1991; Gillies et al., 2005). This is in line with our previous studies demonstrating that selectively targeting α1GABA_A_Rs or nonselectively targeting GABA_A_Rs in the vHipp does not affect dopamine neuron activity (Donegan et al., 2019; Perez et al., 2022). Taken with the findings presented in the current study, it appears that this is due to the targeting of synaptic GABA_A_Rs. However, an open question remains as to why synaptic GABA_A_Rs do not modulate VTA dopamine neuron activity in the way that extrasynaptic ones can. One possibility is that the type of inhibition produced by extrasynaptic receptors (tonic) is more effective at maintaining a decrease in hippocampal activity than synaptic receptors (phasic). It is possible that the fast, transient nature of phasic inhibition is insufficient to produce changes in downstream dopamine activity, whereas the relatively slower and more persistent effects of α5-mediated tonic inhibition have a more robust effect (Koniaris et al., 2011; Schulz et al., 2018). However, this explanation only partially accounts for earlier studies that demonstrate that dampening the excitatory transmission in the vHipp using tetrodotoxin can also restore healthy dopamine system function in animal models used to study psychosis (Lodge and Grace, 2008a; Valenti et al., 2011; Perez and Lodge, 2018).

The loss of efficacy when moved into the synapse may also be explained by a change in receptor functionality. While it has been shown that synaptic α5GABA_A_Rs can successfully contribute to IPSCs (Loebrich et al., 2006; Hausrat et al., 2015; Davenport et al., 2021), differences in the structure caused by loss or gain of protein–protein interactions may prevent GL-II-73 from modulating α5GABA_A_R when they move to the synapse. For example, it is known that α5GABA_A_Rs interact with auxiliary subunits, such as Shisa7, which can modify receptor kinetics (Castellano et al., 2022) and trafficking (Wu et al., 2022) and appear to be critical for tonic currents (Wu et al., 2021). Alterations in protein interactions may change receptor function enough to negate the effects of GL-II-73 in the situation of α5GABA_A_Rs being localized to the synapse.

We confirmed that decoupling α5GABA_A_Rs from radixin did not reduce membrane expression of α5GABA_A_Rs, suggesting that the absence of an effect of GL-II-73 in radixin knockdown rats is not due to a reduction in receptor availability. However, a limitation of this study is that we did not measure actual levels of synaptic and extrasynaptic receptors. Rather, we measured markers of α5GABA_A_Rs localization through association with radixin and gephyrin. Thus, while our coimmunoprecipitation studies suggest that the knockdown of radixin increases synaptic α5GABA_A_Rs, we acknowledge the caveat associated with measuring proxies for localization. Future studies should more rigorously examine the dynamics of α5GABA_A_R relocalization and pinpoint the biological processes resulting in the dramatic loss of efficacy we observed here, despite the incomplete knockdown of radixin. Functional studies demonstrating the impact of radixin knockdown on tonic and phasic GABAergic currents would be helpful, and the absence of these experiments represents a limitation of this study. We emphasize the importance of follow-up studies, as the results obtained here may have important clinical implications, especially as interest in α5-PAMs from the pharmaceutical industry increases. Indeed, α5GABA_A_R localization appears to be dynamically modulated by hippocampal activity levels (Davenport et al., 2021). It is possible that in certain conditions where hippocampal activity is dramatically altered, the proportion of synaptic α5GABA_A_Rs could increase, diminishing the ability of GL-II-73 and perhaps other α5-PAMs as well. However, the results obtained here suggest that this is limited to modulation of dopamine neuron activity, as PPI was unaffected by radixin knockdown. This study highlights the importance of testing novel therapeutics in multiple disease states and/or models, a concept of particular importance for GL-II-73, which has shown promising therapeutic potential for a variety of psychiatric conditions (Prevot et al., 2019, 2020; McCoy et al., 2022; Perez et al., 2022).

## Synthesis

Reviewing Editor: Jibran Khokhar, Western University Department of Anatomy and Cell Biology Decisions are customarily a result of the Reviewing Editor and the peer reviewers coming together and discussing their recommendations until a consensus is reached. When revisions are invited, a fact-based synthesis statement explaining their decision and outlining what is needed to prepare a revision will be listed below. The following reviewer(s) agreed to reveal their identity: NONE. Note: If this manuscript was transferred from JNeurosci and a decision was made to accept the manuscript without peer review, a brief statement to this effect will instead be what is listed below.

This is a well written manuscript that presents novel findings related to positive allosteric modulation of α5GABAARs and how it impacts dopamine signalling in the context of psychosis. The authors have thoroughly addressed the comments raised by previous reviewers.
